# The identification and psychological treatment of panic disorder in adolescents: a survey of CAMHS clinicians

**DOI:** 10.1111/camh.12372

**Published:** 2020-02-23

**Authors:** Holly J. Baker, Polly Waite

**Affiliations:** ^1^ School of Psychology and Clinical Language Sciences University of Reading Reading UK; ^2^ Departments of Experimental Psychology and Psychiatry University of Oxford Oxford UK

**Keywords:** Adolescence, anxiety, cognitive therapy, questionnaires

## Abstract

**Background:**

Panic disorder is experienced by around 1% of adolescents and has a significant impact on social and academic functioning. Preliminary evidence supports the effectiveness of panic disorder‐specific treatment in adolescents with panic disorder; however, panic disorder may be overlooked in adolescents due to overlapping symptoms with other anxiety disorders and other difficulties being more noticeable to others. The aim of this study was to establish what training National Health Service (NHS) Child and Adolescent Mental Health Services (CAMHS) clinicians have received in psychological therapies and panic disorder and how they identify and treat panic disorder in adolescents.

**Method:**

CAMHS clinicians from a range of professions (*n* = 427), who were delivering psychological treatments to children and adolescents with anxiety disorders, participated. They completed a cross‐sectional, online survey, including a vignette describing an adolescent with panic disorder, and were asked to identify the main diagnosis or presenting problem.

**Results:**

Less than half the clinicians (48.6%) identified panic disorder or panic symptoms as the main presenting problem from the vignette. The majority of clinicians suggested CBT would be their treatment approach. However, few identified an evidence‐based treatment protocol for working with young people with panic disorder. Almost half the sample had received no training in cognitive behaviour therapy (CBT), and around a fifth had received no training in delivering psychological treatments.

**Conclusions:**

Only half of CAMHS clinicians identified panic disorder from a vignette and although CBT treatments are widely offered, only a minority of adolescents with panic disorder are receiving treatments developed for and evaluated with young people with panic disorder. There is a vital need for clinician training, the use of tools that aid identification and the implementation of evidence‐based treatments within CAMHS.

**Key Practitioner Message:**

- Panic disorder affects around 1% of adolescents, adversely impacting social, academic and long‐term life functioning.
- There is preliminary evidence for the effectiveness of a panic disorder‐specific treatment of panic disorder in adolescents.
- Clinicians struggle to identify panic disorder or suitable treatment protocols for treating adolescents, although most would use CBT as the treatment approach.
- There is a vital need for clinician training, tools that aid identification of young people with panic disorder and evidence‐based treatments that can be delivered in routine clinical settings.

## Introduction

Panic disorder is characterised by repeated, unexpected panic attacks, involving physical symptoms, such as a racing heart, dizziness and chest pains, along with a fear of recurring attacks and changes in behaviour to avoid further attacks (American Psychiatric Association, 2013). Peak onset of panic disorder is between 15 and 19 years of age (Von Korff, 1985) and is experienced by around 1% of adolescents (Essau, Conradt, & Petermann, 2000; Vizard, Pearce, & Davis, 2018), increasing to around 3% among older adolescents aged between 17 and 19 years (Merikangas et al., 2010; Vizard et al., 2018). There are high levels of comorbidity, with around 90% of adolescents with panic disorder experiencing another anxiety or mood disorder (Birmaher & Ollendick, 2004). It has a significant impact on social and academic functioning and normal development in young people (Kearney, Albano, Eisen, Allan, & Barlow, 1997). Early onset is associated with increased comorbidity with other disorders, increased likelihood of reoccurrence after periods of remission and poorer longer‐term life outcomes (Ramsawh, Weisberg, Dyck, Stout, & Keller, 2011). Left untreated, the condition typically continues into adulthood (Moreau, 1992). Consequently, it is crucial that young people with this debilitating disorder are accurately identified and receive timely, effective treatments.

There appear to be difficulties for clinicians in identifying adolescents with panic disorder, possibly due to overlapping symptoms with other comorbid disorders (especially anxiety disorders) where panic attacks may be common, diagnostic overshadowing (e.g. seeing recurrent panic attacks or behavioural avoidance as being part of another problem, such as generalised anxiety disorder or behavioural difficulties, rather than as panic disorder), or other difficulties being more noticeable and therefore becoming the focus of attention (e.g. difficulties attending school; Alessi, Robbins, & Dilsaver, 1987; Doerfler, Connor, Volungis, & Toscano, 2007). This is highlighted by Doerfler et al. (2007) who examined referrals for 35 young people aged 6–18 years who were referred to a paediatric psychopharmacology clinic, and on assessment, met diagnostic criteria for panic disorder. None of the young people were referred for the treatment of panic disorder or agoraphobia, which occurred in 50% of the sample). Instead, they were referred for an externalising or mood disorder. Once assessed, the most common comorbid externalising conditions were attention deficit hyperactivity disorder (81%) and oppositional defiant disorder (57%), while the most common internalising conditions were separation anxiety disorder (89%) and generalised anxiety disorder (86%).

The most commonly evaluated treatments for a range of anxiety disorders in children and adolescents are psychological interventions, in particular, cognitive behaviour therapy (CBT; Warwick et al., 2017). Although there are a large number of studies evaluating a generic form of CBT [e.g. ‘Coping Cat’ (Kendall, 2006), ‘C.A.T. Project’ (Kendall, 2002)] for young people with a range of anxiety disorders, very few studies include young people with panic disorder and where they do, specific outcomes for adolescents with panic disorder are unknown. In a meta‐analysis of CBT for anxiety disorders in children and adolescents, James, James, Cowdrey, Soler, and Choke (2013) found a remission rate of 59% post‐treatment. However, of the 41 randomised controlled studies included, only six included young people with panic disorder (Barrington, Prior, Richardson, & Allen, 2005; Chalfant, Rapee, & Carroll, 2007; Cobham, 2012; Hudson et al., 2009; Nauta, Scholing, Emmelkamp, & Minderaa, 2003; Sung et al., 2011) and young people with panic disorder accounted for only 2%–9% of the total samples.

There is preliminary evidence for the effectiveness of a panic disorder‐specific CBT treatment for adolescents aged 11–17 years (Hoffman & Mattis, 2000), using the manualised treatment protocol, 'Mastery of Anxiety and Panic for Adolescents: Riding the Wave' (MAP‐A) (Pincus, Ehrenreich, & Mattis, 2008). In a small randomised feasibility study, 13 adolescents aged 14–17 years who received 11 weekly sessions of MAP‐A showed significantly greater reductions in clinician severity ratings of panic disorder than controls, with a large effect size (*d* = 1.09; Pincus, May, Whitton, Mattis, & Barlow, 2010). In a randomised study among 11 to 17 year‐olds (*n* = 54), of an intensive version of the treatment, conducted over eight consecutive days, significantly greater reductions in panic severity were found at six weeks post‐treatment in the MAP‐A group compared with the wait list control group [*F* (1, 37) = 11.48, *p* < .01; Elkins, Gallo, Pincus, & Comer, 2016].

Due to the limited evidence to date, there is a lack of guidance for how clinicians should treat panic disorder in young people. The U.K. National Institute for Health and Care Excellence (NICE) makes no recommendations for assessing or treating children and adolescents with panic disorder, although for adults, the recommended treatment is 7–14 hr of weekly CBT sessions (NICE CG22 and CG113, NICE, 2011).

Given the significant burden that panic disorder presents in adolescence and into adulthood, successful early identification and treatment within routine clinical services are crucial. The preliminary evidence supporting the use of a panic disorder‐specific treatment in adolescents suggests there is clinical utility in using a diagnostic framework (e.g. DSM‐5, American Psychiatric Association, 2013) to make sense of the young person’s difficulties and guide treatment decisions.

As we currently know very little about whether panic disorder is being identified or how it is being treated within routine services, the aim of this study was to survey National Health Service (NHS) Child and Adolescent Mental Health Services (CAMHS) clinicians in England to establish (a) what training clinicians have had in delivering psychological therapies and panic disorder, (b) if clinicians identify panic disorder/symptoms from a clinical vignette describing an adolescent with panic disorder, (c) whether there are significant differences in the identification of panic disorder/symptoms depending on clinicians’ professional backgrounds, (d) what treatments are currently delivered in CAMHS for panic disorder, and (e) what were clinicians’ experiences of working with adolescents with panic disorder.

## Method

### Design

The survey used a cross‐sectional, self‐report questionnaire design. Ethical approval for this study was granted by the School of Psychology & Language Science Ethics Committee at the University of Reading. Approval was also granted by the Health Research Authority (HRA; reference: 18/HRA/1564) to conduct the study with (NHS) staff.

### Participants

To be eligible to participate, clinicians had to be currently working clinically in NHS CAMHS in England and delivering psychological treatments to children and adolescents with anxiety disorders. Clinicians came from 51 NHS Healthcare Trusts (organisations within the NHS providing healthcare services across broad designated geographical areas), from all 15 National Institute for Health Research (NIHR) Local Clinical Research Network (LCRN) areas and included 46 out of the 48 counties in England, covering both urban and rural areas of England. A total of 427 participants completed the survey. A broad range of CAMHS professionals participated, including clinical psychologists, psychiatrists and nurses. See Table 1 for further information about sample characteristics.

**Table 1:** Characteristics of CAMHS clinicians (*n* = 427)

Characteristic	% (*n*)	Mean (standard deviation), range
Gender		
Female	78.7 (336)	–
Age		
18–25	7.0 (30)	–
26–35	32.1 (137)	–
36–45	26.5 (113)	–
46–55	24.6 (105)	–
56–65	9.1 (39)	–
66+	0.7 (3)	–
Professional background		
Community psychiatric nurse	22.2 (95)	–
Clinical psychologist	20.8 (89)	–
Psychiatrist	11.9 (51)	–
Social worker	9.4 (40)	–
Psychotherapist	8.9 (38)	–
Trainee^a^	5.2 (22)	–
Occupational therapist	4.4 (19)	–
Nurse^b^	3.8 (16)	
Assistant psychologist	3.7 (16)	–
Psychological well‐being practitioner	1.9 (8)	–
Counsellor	1.2 (5)	–
CBT therapist	1.4 (6)	
Other^c^	5.2 (22)	–
Experience and working practice		
Years of clinical practice	–	*M* = 10.02 (*SD* = 9.78), <1–40
Years employed in current trust	–	*M* = 6.84 (*SD* = 7.29), <1–40
Employed full‐time in CAMHS	68.6% (293)	–

a ‘Trainees’ were training in clinical psychology (*n* = 7, 1.6%), CBT (*n* = 5, 1.2%), children’s well‐being practitioner (*n* = 5, 1.2%), psychiatrist (*n* = 2, 0.5%), play therapist (*n* = 1,0.2%), mental health nurse (*n* = 1, 0.2%) and ‘CYP IAPT’ trainee (*n* = 1, 0.2%).

b Nurses were mental health, learning disability and general nurses.

c ‘other’ were art therapists (*n* = 3, 0.7%), systemic practitioners (*n* = 4, 0.9%), support workers (*n* = 5, 1.2%), interpersonal therapist (*n* = 1, 0.2%), forensic psychologist (*n* = 1, 0.2%), speciality and associate specialist doctor (*n* = 1, 0.2%), counselling psychologist (*n* = 3, 0.7%).

### Recruitment

Participants were recruited through NHS Healthcare trusts in England, either directly or via the LCRNs. LCRNs emailed healthcare trusts within their respective regions and encouraged participation. Trusts were also contacted directly via email by the first author, and if they agreed to participate, they distributed an invitation email to CAMHS clinical staff, along with an information sheet containing a link to the online consent form and questionnaire. Because we wanted to examine how well clinicians were able to identify panic disorder, study materials described the study in broad terms and referred to anxiety in adolescents, rather than specifically mentioning panic disorder. A reminder email was sent to clinicians 1–2 weeks after the first invitation. Some participating trusts also promoted clinician participation at team meetings and on staff intranet systems. Participants were offered the opportunity to enter a prize draw to win one of two £100 Amazon vouchers as an incentive to participate. Recruitment took place between 1 April and 31 December 2018.

### Procedure

Once participants had read the information sheet and given informed consent, they completed an online self‐report questionnaire, which took approximately 10–15 min to complete. Paper copies of the survey were available on request. Although participants gave their name as part of the consent process and were asked to provide details of the NHS Trust that employed them for NIHR reporting purposes, survey responses were anonymised by allocation of a unique identification number created at the time of participation. Identifying information was removed from the data set and the data unlinked so that responses were anonymous.

### Measures

A questionnaire was designed specifically for this study (see Appendix S1). Participants were not informed that the study was specifically about panic disorder but were asked to participate in a study about anxiety disorders more broadly among adolescents in CAMHS. Participants provided demographic information and details of their professional background and experience. They then read a clinical vignette of a 15‐year‐old girl and were asked to suggest (a) what they thought the main diagnosis or primary presenting problem was and (b) what treatment she should be offered. On the basis of the information provided in the vignette, the girl met DSM‐5 criteria for a diagnosis of panic disorder (American Psychiatric Association, 2013) and reflected how a young person with panic disorder typically presents (i.e. with a history of other anxiety difficulties). The symptoms described did not fulfil criteria for any other DSM‐5 diagnosis. Only after completing the vignette questions, were clinicians then asked questions explicitly related to panic disorder in young people. This included questions about their experience of working with young people with panic disorder, how panic disorder is treated in CAMHS, their training in delivering psychological therapies and perceived training needs. Participants were required to complete all required questions in each section before being able to move to the next section and were unable to go back to previous sections ensuring earlier responses could not retrospectively be amended. Clinicians provided demographic information and details about their professional background, years spent as a qualified/practicing clinician and information about their service through both open and closed questions. Questions about the primary presenting issue or diagnosis for the vignette, known guidelines or protocols for both the vignette and working with young people with panic disorder, the age of young people first presenting with panic symptoms or disorder, the nature of their difficulties, clinicians’ training needs and available funding for training were all provided as open questions. Questions related to clinicians’ recommended treatment approach for the vignette, recommended number of sessions, whether they would work primarily with the young person and/or parents/carers, their experience in working with young people with panic disorder and their previous training in working with young people with DSM‐5 anxiety disorders were all provided as closed questions. Free text boxes were provided in some instances in order to provide more information, that is to elaborate on a response or to provide information if none of the preselected answers were applicable and the clinician had selected ‘other’. The vignette and questions were developed in collaboration with clinicians (PW and other clinical psychologists specialising in child and adolescent anxiety disorders within the research group), and members of the Anxiety and Depression in Young People (AnDY) Research Advisors Group (young people with lived experience of anxiety and depression). The questionnaire was piloted by a clinical psychologist specialising in child and adolescent mental health, and adaptations were made.

### Sample size

A priori precision‐based sample size calculation was carried out (http://epitools.ausvet.com.au), which indicated that 385 clinicians were required for 0.05 precision level, 95% confidence interval and 5% margin of error based on an infinite population. Consequently, with a final sample of 427, we are able to generalise our results confidently to the wider population of CAMHS clinicians.

### Data analysis

For each question, frequencies were examined, producing proportions of clinician’s responses in percentages. All frequencies and percentages were based on completer data. For open‐ended questions, a coding system was developed by the authors in line with the responses received. This was then piloted on a subsample and refined before the full data set was then independently double‐coded by the authors. Agreement between coders was high (*K* = .76 to *K* = 1.00). Where discrepancies arose, this was discussed in order to come to an agreement. Survey responses were analysed using IBM SPSS (Version 24). To examine differences in the identification of panic disorder for the clinical vignette between professional groups, qualified clinicians with a core profession were classified into three groups, which were defined on the basis of the professional backgrounds of the participants: (a) professionals trained in psychological therapies using diagnostic frameworks (clinical psychologists and psychiatrists), (b) healthcare/social care professionals not primarily trained in psychological therapies (nurses, occupational therapists and social workers) and (c) professionals primarily trained in psychological therapies (counsellors, counselling psychologists, psychological well‐being practitioners, CBT/IPT/art therapists, systemic practitioners and psychotherapists). We examined differences in identification rates between these groups using a chi‐square analysis and post hoc pairwise comparisons.

## Results

### Clinicians’ professional backgrounds and training

The percentage of participants who had received training in CBT was 53.6% (*n* = 229) and 35.3% (*n* = 151) gave further information about the nature of this training; of these participants, 18.5% (*n* = 28) had completed the training as part of their professional qualification (e.g. DClinPsy), 43% (*n* = 65) had completed a one‐year postgraduate training course in CBT (unspecified whether for working with children and young people or adults), 11.3% (*n* = 17) had completed a one‐year postgraduate training course in CBT specifically for children and young people and 6% (*n* = 9) specifically for adults. A fifth (*n* = 32, 21.2%) had received CBT training as part of a workshop of between 1 and 5 days. Of the 46.4% of participants (*n* = 198) who had not trained in CBT, just under a quarter (*n* = 100, 23.4%) had trained in non‐CBT treatment approaches (e.g. systemic family therapy or psychotherapy), while 19.0% (*n* = 81) had not received any training in the delivery of psychological treatments and 4% (*n* = 17) did not specify. When asked about training in working with young people with anxiety disorders, 61.8% (*n* = 264) of clinicians had received training in the treatment of one or more of any DSM‐5 anxiety disorders and 41.2% of clinicians (*n* = 176) had received training on panic disorder in children and young people. When asked about what training clinicians felt they needed 60.9% responded (*n* = 260), and 33% of these (*n* = 87) wanted training in psychological treatment approaches generally, and 20% (*n* = 51) specifically wanted CBT training. More than half of all clinicians who participated (*n* = 250, 58.5%) indicated that they would like to receive training in panic disorder in young people.

### The identification of panic disorder

When presented with the vignette of an adolescent with symptoms consistent with a DSM‐5 diagnosis of panic disorder, less than half the clinicians identified panic disorder (*n* = 187, 43.8%) or panic symptoms (*n* = 21, 4.9%) as the primary diagnosis or presenting problem. A further 11.2% (*n* = 48) identified panic symptoms as secondary, with another anxiety disorder as primary. Table 2 provides details of all primary diagnoses or problems suggested by clinicians.

**Table 2:** Clinicians’ suggestions of primary diagnoses/presenting problem (*n* = 421), treatment approach (*n* = 427) and number of sessions required (*n* = 410) for the clinical vignette

	% (*n*)
Diagnosis or primary presenting problem	
Panic disorder	43.7 (187)
Panic symptoms	4.9 (21)
Anxiety (unspecified) with panic	4.9 (21)
Generalised anxiety disorder with panic	3.7 (16)
Social anxiety disorder with panic	1.9 (8)
Separation anxiety disorder with panic	0.2 (1)
Agoraphobia	0.5 (2)
Anxiety (unspecified)	22.0 (94)
Generalised anxiety disorder	7.0 (30)
Social anxiety disorder	6.1 (26)
Separation anxiety disorder	2.3 (10)
Nonanxiety disorder	1.2 (5)
Treatment approach	
Cognitive behaviour therapy	87.1 (372)
Brief solution focused therapy	4.4 (19)
Family therapy	3.0 (13)
Psychotherapy	2.1 (9)
Other (specified)^a^	1.6 (7)
Other (unspecified)	1.6 (7)
Number of sessions to be offered	
<4	0.9 (4)
4–8	38.6 (165)
9–12	46.1 (197)
13–20	11.2 (48)
21+	1.4 (6)

a ‘Other’ treatment approaches were psychoeducation (*n* = 3, 0.7%), art therapy (*n* = 2, 0.5%), narrative approach (*n* = 1, 0.2%) and anxiety group work (*n* = 1, 0.2%).

Table 3 gives the percentage and number of clinicians from each professional background who identified panic disorder or panic symptoms as the primary presenting problem. When we compared responses from qualified clinicians from different professional backgrounds, there was a significant association between participants’ profession and the likelihood of identifying panic disorder or panic symptoms as primary (*χ* ^2^ (2) *= *34.712, *p* = .00), with 69.6% of Group 1 (clinical psychologists and psychiatrists, *n* = 138), 40.8% of Group 2 (nurses, social workers and occupational therapists, *n* = 169) and 32.8% of Group 3 (psychotherapists, counsellors, counselling psychologists, CBT/IPT/art therapists, systemic practitioners and psychological well‐being practitioners, *n* = 67), identifying primary panic disorder/symptoms. Post hoc pairwise comparisons using Bonferroni adjustment found professionals trained in diagnostic frameworks (Group 1) were significantly more likely to identify panic disorder compared to Group 2 (*χ* ^2^ (1) *= *25.235, *p* = .00) and Group 3 (*χ* ^2^ (1) *= *24.908, *p* = .00). There were no significant differences between Groups 2 and 3 (*χ* ^2^ (1) = 1.294, *p* = .255).

**Table 3:** The percentage and number of clinicians from each professional background who identified panic disorder or panic symptoms as the main presenting problem in the clinical vignette (*n* = 421)

Professional background	Total *n* = 421	% (*n*) identifying panic disorder/symptoms
Clinical psychologist	89	69.7 (62)
Psychiatrist	49	69.4 (34)
Trainee^a^	20	60.0 (12)
Occupational therapist	18	50.0 (9)
Community psychiatric nurse	95	40.0 (38)
Social worker	40	40.0 (16)
Psychotherapist	38	39.5 (15)
Psychological well‐being practitioner	8	37.5 (3)
Counsellor	5	0.0 (0)
Counselling psychologist	2	0.0 (0)
Other	57	–
Forensic psychologist	1	100.0 (1)
Support worker	5	80.0 (4)
No core profession	4	75.0 (3)
CBT therapist	6	66.7 (4)
Assistant psychologist	16	62.5 (10)
Nurses^b^	16	37.5 (6)
Systemic practitioner	4	0.0 (0)
Art therapist	3	0.0 (0)
Interpersonal therapist	1	0.0 (0)
Speciality and associate specialist doctor	1	0.0 (0)

a ‘Trainees’ were training in clinical psychology (*n* = 7, 1.6%), CBT (*n* = 5, 1.2%), children’s well‐being practitioner (*n* = 5, 1.2%), psychiatrist (*n* = 2, 0.5%), play therapist (*n* = 1,0.2%), mental health nurse (*n* = 1, 0.2%) and ‘CYP IAPT’ trainee (*n* = 1, 0.2%).

b Nurses were mental health, learning disability and general nurses.

### The treatment of panic disorder

As shown in Table 2, when clinicians were asked what their treatment approach would be, 87.1% (*n* = 372) said they would offer CBT, with almost half the sample (*n* = 197, 46.1%) suggesting between 9 and 12 treatment sessions. When asked if they were aware of any treatment protocols appropriate to use for the clinical case, just 1.2% of clinicians (*n* = 5) identified a protocol that had been evaluated for young people with panic disorder (i.e. ‘MAP‐A’, Pincus et al., 2008). A further 1.4% (*n* = 6) identified a transdiagnostic CBT protocol that had been evaluated for young people with a range of anxiety disorders (including a small number with panic disorder; i.e. Kendall, 2006; Lyneham, Abbott, Wignall, & Rapee, 2003). A panic disorder‐specific protocol designed for use with adults (i.e. Barlow & Craske, 1988; Clark, & Salkovskis, 2009) was identified by 10.5% of clinicians (*n* = 45) and 11.5% (*n* = 49) suggested a nonspecific CBT protocol for anxiety disorders (e.g. ‘CBT protocol’). Almost half of clinicians (*n* = 201, 47.1%) did not identify a suitable treatment protocol, and a further 28.3% (*n* = 121) gave a nonprotocol answer, such as ‘CYP IAPT guidelines’, ‘NICE guidelines’ or ‘local NHS guidelines’.

When clinicians were asked specifically about how they would treat young people with panic disorder, the pattern of responses was broadly similar; 0.9% (*n* = 4) of clinicians indicated they would use Pincus et al.'s MAP‐A, 6.6% (*n* = 28) suggested a transdiagnostic CBT protocol, 7.5% (*n* = 32) identified a panic disorder‐specific protocol designed for adults and the remaining 85% (*n* = 363) did not identify a suitable protocol.

### Clinicians' experience of young people with panic disorder in CAMHS

When clinicians were asked about their experience of working with young people with panic disorder, the majority (*n* = 354, 82.9%) reported they saw young people with panic disorder within their CAMHS service. Over two thirds of participants (*n* = 293, 68.6%) reported that they had personally treated panic disorder in their current role, while 26.5% (*n* = 113) said they had not and 4.9% (*n* = 21) were unsure. Of those clinicians who responded to further questions about how young people with panic disorder presented in services (*n* = 305, 71.4%), around two thirds (*n* = 195, 63.9%) believed young people with panic disorder were entering CAMHS as adolescents aged 12–18 years. However, a third (*n* = 102, 33.4%) felt they were seeing young people with panic disorder starting at primary school age (7–11 years), with a further eight (2.6%) clinicians believing they were seeing young people with panic disorder below the age of 7 years.

## Discussion

This national survey of CAMHS clinicians provides an insight into the training backgrounds and needs of clinicians, whether clinicians identified panic disorder/symptoms in an adolescent (and whether this significantly differed by professional background), how children and young people with panic disorder are currently treated in services and clinicians’ experiences of working with young people with panic disorder. The main findings were that less than half of clinicians identified panic disorder or panic symptoms as being the main presenting problem in a clinical vignette where the young person met DSM‐5 criteria for a diagnosis of panic disorder (and no other disorders). While the majority of clinicians suggested CBT should be the main treatment approach, nearly half the clinicians had not received any training in CBT and a fifth had not had *any* therapy training. Additionally, very few identified an evidence‐based treatment protocol for working with young people with panic disorder.

Our results revealed that although two thirds of clinicians reported they were working clinically with young people with panic disorder, less than half identified panic disorder or panic symptoms as the primary diagnosis or presenting problem from a clinical vignette, and the identification of panic disorder or symptoms was influenced by professional background. If clinicians are not able to identify a young person’s main presenting problem, it would appear unlikely that they are then delivering a specific, evidence‐based treatment, such as MAP‐A (Pincus et al., 2008). Nevertheless, this is consistent with the findings of Doerfler et al. (2007) where panic disorder may not be identified in young people. In our survey, clinicians frequently misidentified the young person’s main difficulty as generalised or social anxiety disorder, again consistent with the existing literature (Achiam‐Montal, Tibi, & Lipsitz, 2013; Doerfler et al., 2007; Masi, Favilla, Mucci, & Millepiedi, 2000). Clinicians from professional backgrounds that involved training in diagnostic frameworks (i.e. clinical psychologists and psychiatrists) were significantly more likely to identify panic disorder/symptoms than practitioners from allied health/social care backgrounds or those trained primarily as therapists. However, still nearly a third of clinical psychologists and psychiatrists did not identify panic symptoms/disorder as the primary problem. Practitioners from allied health/social care backgrounds or those trained primarily as therapists may be less likely to identify panic disorder or symptoms due to the use of a therapeutic approach that focuses on the phenomenological experience of the client, making them reluctant to engage with the medical model due to concerns around stigma and labelling (Larsson, Brooks, & Loewenthal, 2012). It was also notable that, although sample sizes were small, some professional groups (e.g. support workers) had high rates of identification of panic disorder or symptoms, despite the use of diagnostic frameworks not being part of their core training. It is possible that the context in which they work influences the way they conceptualise a young person’s difficulties. Although the majority of CAMHS clinicians would offer CBT in the case of the clinical vignette, only 13.1% identified either an evidence‐based panic disorder‐specific protocol or a generic treatment protocol for young people with a range of anxiety disorders including panic disorder. It is possible that this relates to significant gaps in the evidence base for treatment. Very few studies evaluating generic, transdiagnostic approaches include adolescents with panic disorder, and when they are included, they make up less than 10% of the sample and their specific outcomes have not been investigated. Around 10% of clinicians identified a panic disorder‐specific protocol designed and evaluated with adults, for example cognitive therapy based on Clark’s model of panic (Clark, 1986, 1994, 1999). Although this treatment approach is highly effective in treating panic disorder in adults, it has not been evaluated with adolescents and therefore its use at this stage may be premature.

This study suggests that there continue to be significant skills shortages and training needs within CAMHS. It is well recognised that professionals working with children and young people with mental health difficulties must have the necessary competencies, skills and training in order to identify the needs and improve the outcomes of the young people they work with (DoH, 2015; Hargrave, 2004). Over a decade ago, Stallard, Udwin, Goddard, and Hibbert (2007) found that while CBT was the dominant treatment approach used by CAMHS clinicians, around a third rated their CBT skills as ‘inadequate or fairly basic’ and two thirds identified child‐focused CBT as a training need (Stallard et al., 2007). This study suggests that these issues have not been adequately addressed in the intervening period and that the training needs of CAMHS practitioners must be prioritised in the future (Edwards, Williams, Dogra, O'Reilly, & Vostanis, 2008).

### Implications for researchers, training providers and clinical services

The current study suggests that there is an urgent need to develop the skills of the current CAMHS workforce, within core and postqualification training and continued professional development activities. As training and development can be resource‐intensive, the development and evaluation of training programmes that can easily be disseminated to clinicians at scale (e.g. using online resources) are vital. Further research is required to improve the identification and treatment of young people with panic disorder. A first step would be to develop and evaluate assessment measures to be used within CAMHS, where due to time constraints, use of diagnostic interview schedules may be unrealistic. Given there is preliminary evidence for the effectiveness of a panic disorder‐specific treatment for adolescents (Hoffman & Mattis, 2000; Pincus et al., 2010), we would encourage clinicians to use this approach within services. However, as there is only one small RCT supporting the use of this treatment in a format that could be easily delivered in CAMHS (i.e. not involving intensive treatment over consecutive days), research is required to develop the evidence base for treatments that can be delivered within routine clinical services. As transdiagnostic treatments appear to be used with young people with panic disorder, examination of whether these treatments are effective in treating panic disorder in young people specifically is important and to compare them to panic disorder‐specific treatments. As around 10% of clinicians are using protocols designed for use with adults (e.g. Clark’s cognitive therapy), adapting and evaluating this approach for use with adolescents may also be worthwhile.

### Strengths and limitations of the current study

This study was conducted with a large number of clinicians from a wide range of professional disciplines, broadly in line with reports of the CAMHS workforce in that around a third of clinical staff were nurses, a third were clinical psychologists and psychiatrists, with the remaining third made up of other professional groups. This is consistent with Barnes, Devanney, Uglebjerg, Wistow, and Hartley (2010), where nurses made up the largest professional group (23%), followed by psychologists (10%) and psychiatrists (12%). With participation from clinicians within 51 NHS trusts within England, covering 46 out of the 48 counties in England, it provides a nationally representative picture. The survey was designed with input from experienced clinicians and service users, increasing validity. Nevertheless, as the survey was distributed by NHS trusts and CAMHS services, we lack data on nonresponders. Although these results provide a good insight into CAMHS, many young people do not reach the required thresholds for CAMHS (Crenna‐Jennings & Hutchinson, 2018) and are likely to be seen in other non‐NHS mental health services provided by local authorities (government organisations responsible for delivering regional public services) and voluntary sector organisations (e.g. counselling services). It will be of importance to understand what is happening in these wider services in the future. Although our findings give a good insight into NHS CAMHS in England, we cannot generalise our findings to other countries and highlight the ongoing need to gain an understanding of the international picture.

## Conclusion

Panic disorder is a disabling condition that left untreated persists into adulthood with far‐reaching personal, social and economic implications. Less than half of the participating CAMHS clinicians identified panic disorder or panic symptoms from a clinical vignette. Although CBT was widely used, clinicians struggled to identify protocols that have been demonstrated to be effective either with young people with panic disorder or a range of anxiety disorders including panic disorder, or in adults with panic disorder. There is a vital need for clinician training, tools that aid the identification of young people with panic disorder and evidence‐based treatments that can be delivered effectively in routine clinical settings.

## Ethical information

Ethical approval for this study was granted by the School of Psychology & Language Science Ethics Committee at the University of Reading. Approval was also granted by the Health Research Authority (HRA) (reference: 18/HRA/1564) to conduct the study with NHS staff. Written consent was obtained from the subjects, as appropriate.

## Supporting information


**Appendix S1** **.** Questionnaire for participants.
