# Supplementary material for: The identification and psychological treatment of panic disorder in adolescents: a survey of CAMHS clinicians
Source: Child Adolesc Ment Health. 2020 Feb 23;25(3):135–42. doi: 10.1111/camh.12372 (PMC7496854; doi:10.1111/camh.12372)
Supplement: Supplementary file 1 — Appendix S1 . Questionnaire for participants. [file CAMH-25-135-s001.docx]

Identification and treatment of panic disorder in adolescents

**Supporting information – The identification and psychological treatment of panic disorder in adolescents: a survey of CAMHS clinicians – by Baker & Waite**

**Appendix S1.** Questionnaire for participants.


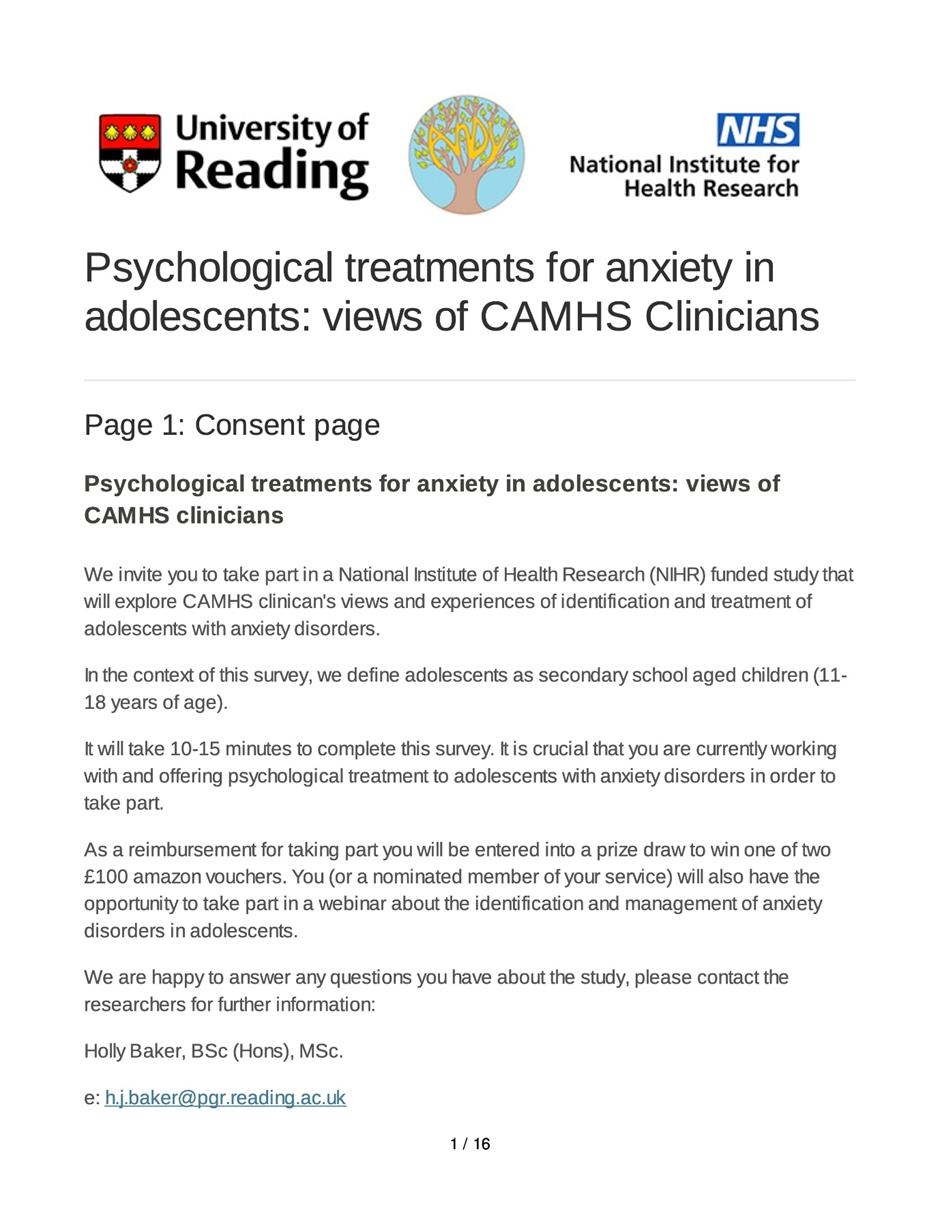


1

Identification and treatment of panic disorder in adolescents


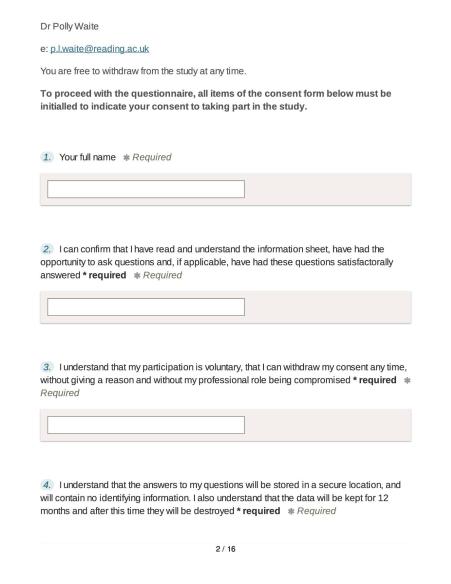


2

Identification and treatment of panic disorder in adolescents


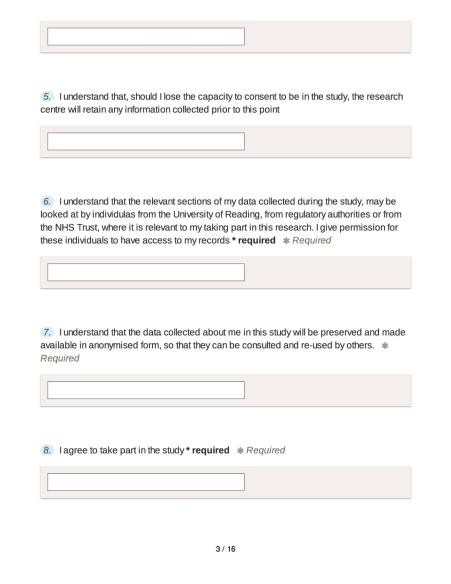


3

Identification and treatment of panic disorder in adolescents


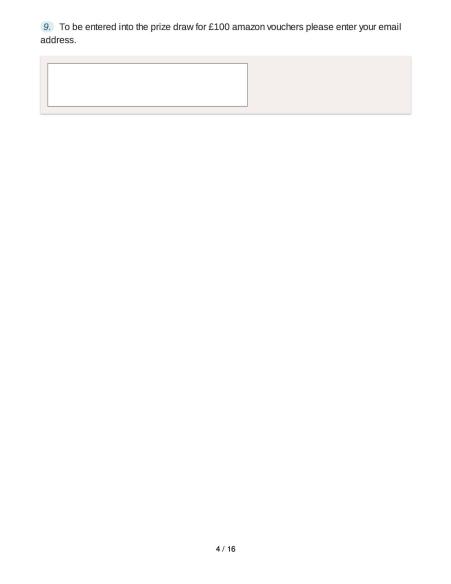


4

Identification and treatment of panic disorder in adolescents


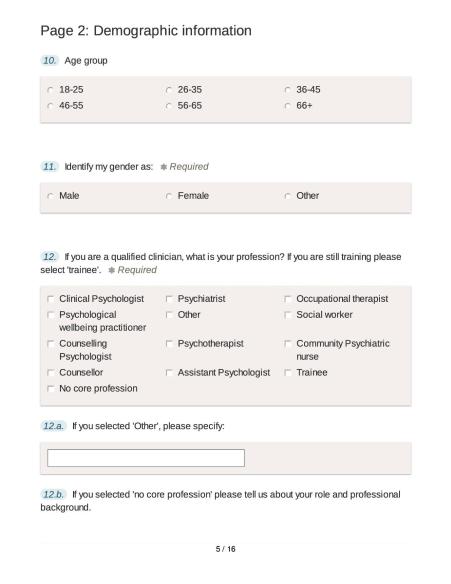


5

Identification and treatment of panic disorder in adolescents


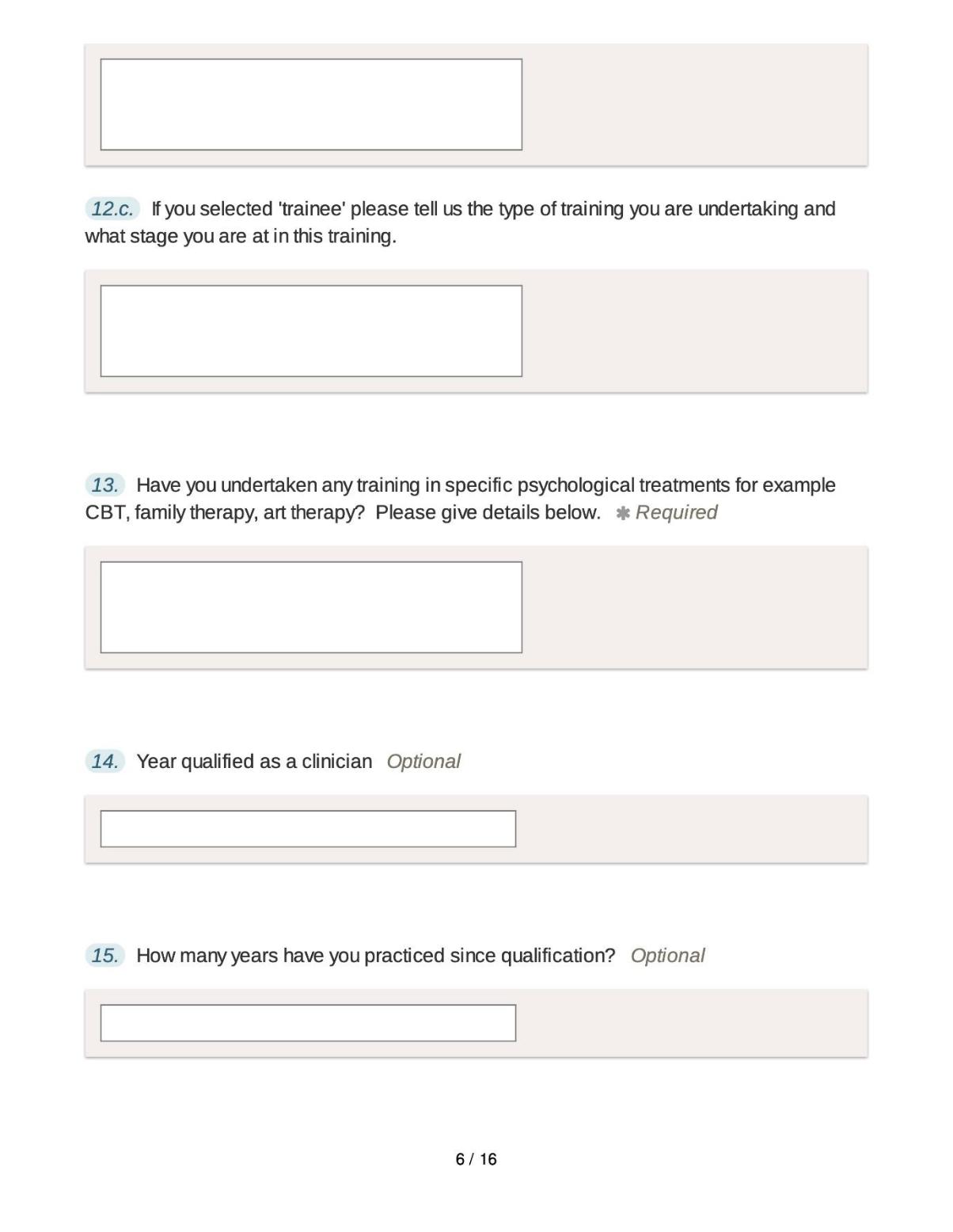


6

Identification and treatment of panic disorder in adolescents


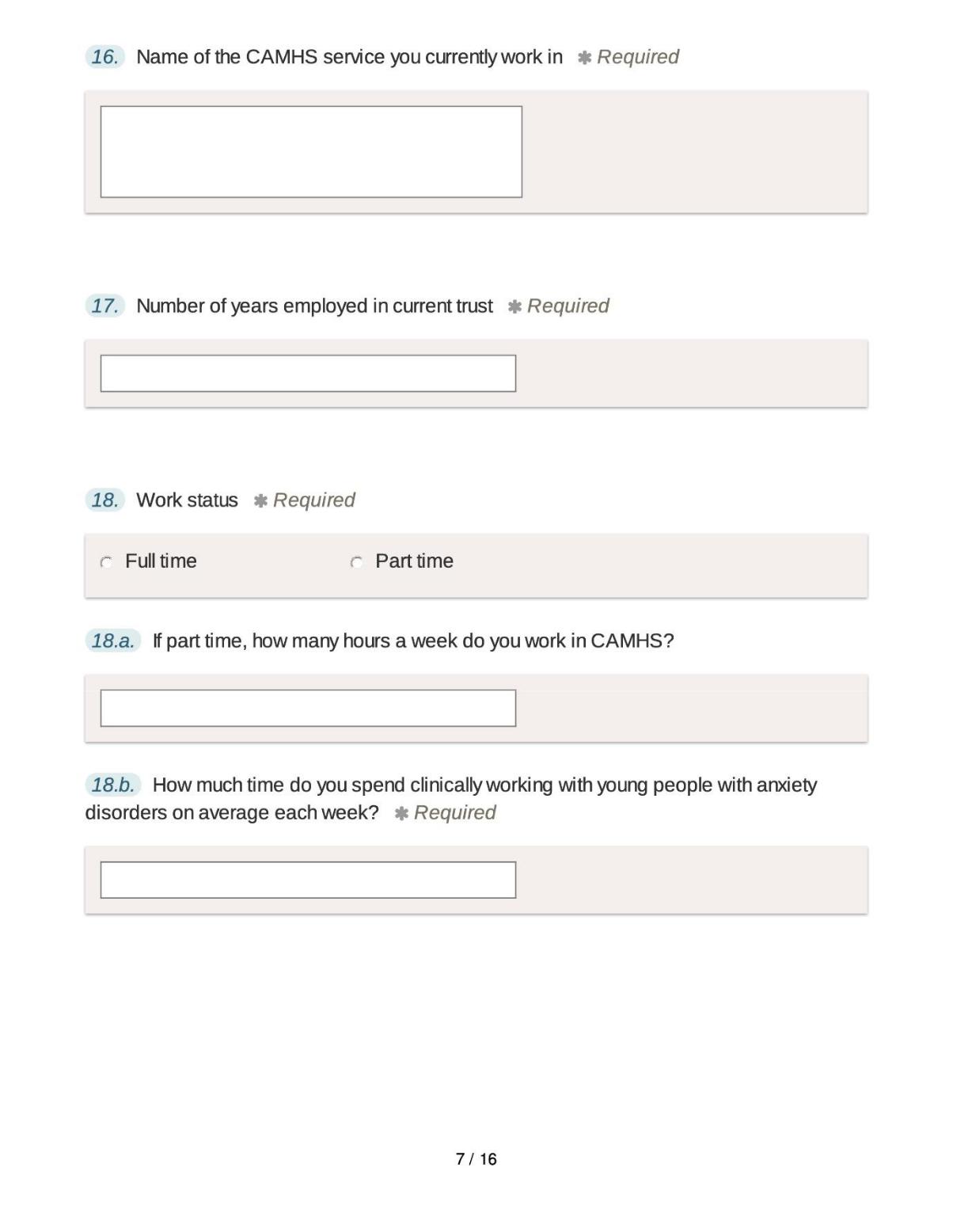


7

Identification and treatment of panic disorder in adolescents


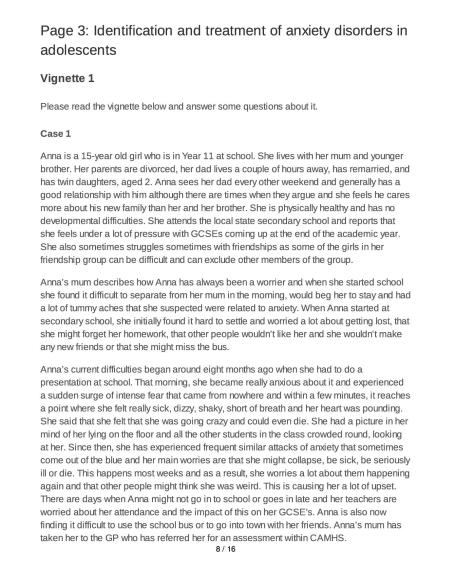


8

Identification and treatment of panic disorder in adolescents


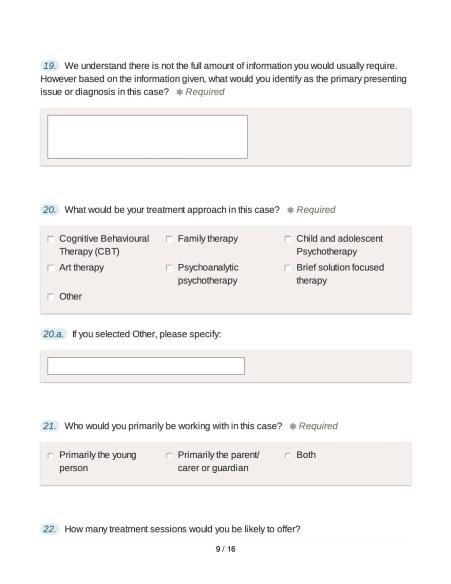


9

Identification and treatment of panic disorder in adolescents


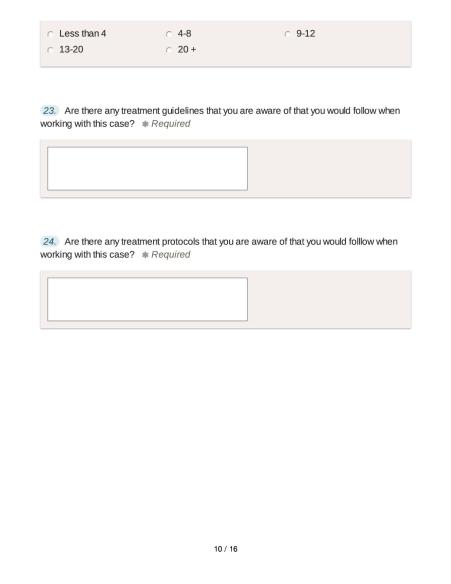


10

Identification and treatment of panic disorder in adolescents


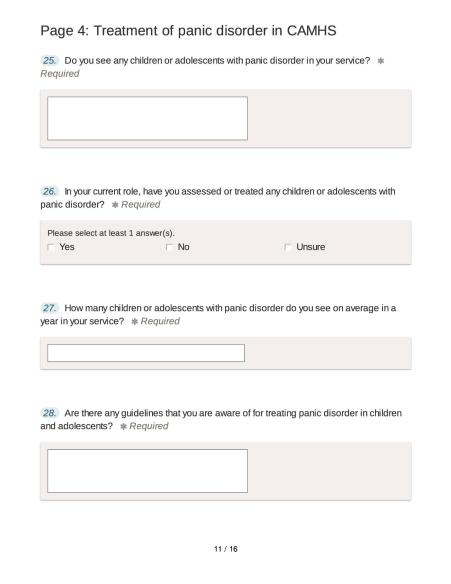


11

Identification and treatment of panic disorder in adolescents


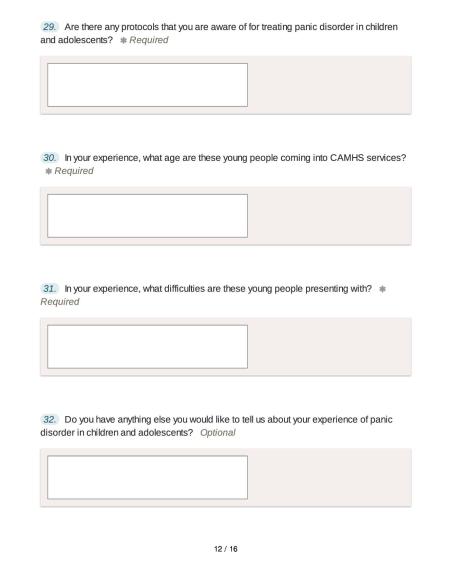


12

Identification and treatment of panic disorder in adolescents


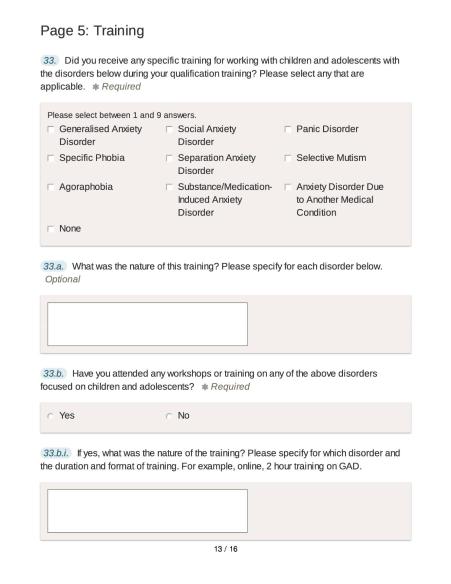


13

Identification and treatment of panic disorder in adolescents


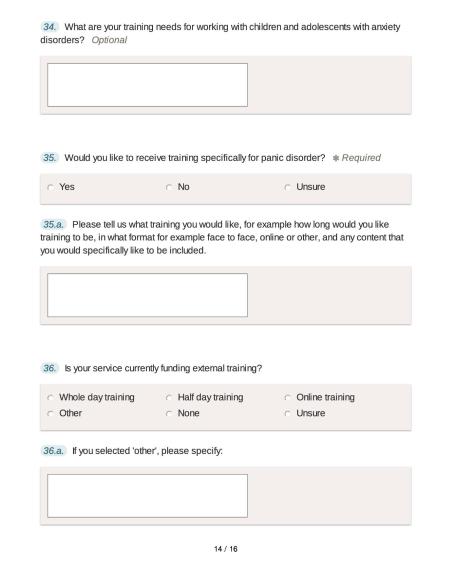


14

Identification and treatment of panic disorder in adolescents


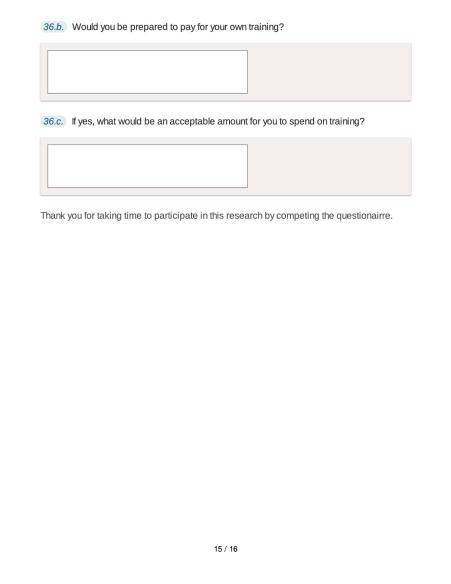


15

Identification and treatment of panic disorder in adolescents


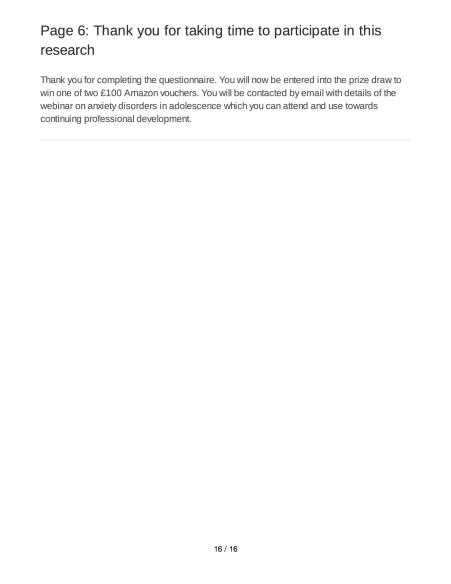


16
